# When trabeculectomy fails

**Published:** 2012

**Authors:** Ian Murdoch

**Affiliations:** Senior lecturer and consultant ophthalmologist, Department of I Epidemiology and International Eye Health, Institute of Ophthalmology, London, UK.

**Figure:**
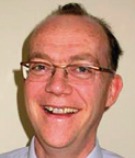
Ian Murdoch

The patient needed the trabeculectomy and you have done a great surgical job, but the pressure is now above the pre-operative level and you are feeling a failure. What are you going to do? What follows is a personal suggestion of questions to ask in developing a strategy.

## How long is it since the operation?

If within three months of the operation, then routine post-operative management should be employed. During this period elevated intraocular pressure (IOP) develops in some patients. This occurs as early as four weeks in some African ethnic groups but is more typically at about six to eight weeks post-operatively. It is part of the wound remodelling process and resolves with good subsequent operative results.^1^ The difficulty lies in telling it apart from frank scarring and failure which has to be addressed with every tool available (page 73).

After three months, it is safe to say you have a fully-developed failure on your hands!

## Is there residual function in the trabeculectomy?

It is always crucial to remember the purpose of the trabeculectomy, namely to prevent ongoing glaucomatous optic nerve damage. Occasionally the recorded IOP post-trabeculectomy differs little from the pre-operative pressure and yet progression of glaucoma is halted.

Nothing need be done in this case! All-day pressure readings post-trabeculectomy frequently show remarkably stable pressures, which is generally not the case when glaucoma is progressing. This may partly explain the apparent paradox.

Another option is to simply restart ocular hypotensive therapy. This is frequently sufficient, as evidenced by the ‘partial success’ figures in all trabeculectomy surgical trial outcomes. To optimise the topical therapy, remember to carefully explore the past records for the drug group that was best tolerated and most effective prior to the procedure.

**Figure 1:**
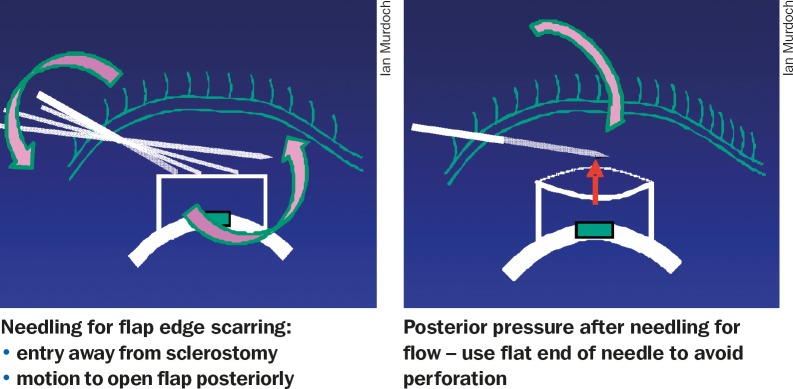
Approach to needing if site of scarring at flap edge

## Is the environment working against success?

The environment may be local concurrent ocular disease such as uveitis, inflamed conjunctiva from allergy or blepharitis, lid pathology, or past ocular surgery. All of these should be treated first or a suitable strategy developed to minimise any subsequent procedure being at risk of failure from this secondary pathology.

The adverse environment may equally include external factors. Poor social situations for post-operative care include living alone and being unable to instil eye drops, or personal well-being having lower priority than child care and other responsibilities. Facilitate an improved environment wherever possible. Ask relatives to administer therapy. Help the patient to find a more satisfactory post-operative management environment. Consider depo-steroid use rather than intensive eye drop regimens. Consider review times that match the other time constraints for the patient.

**‘As with any procedure, the results in your hands are what matter, not the minutiae of the technique compared to someone else’**

## Is the trabeculectomy amenable to ‘resuscitation’?

This involves careful examination with gonioscopy to ensure the sclerostomy is patent and free from internal obstruction. The conjunctival mobility, inflammation, and vascularisation should be noted. When examining the bleb, the most frequent site of failure is either scarring at the edge of the scleral flap with flat overlying tissues, or else encapsulation of the trabeculectomy flap with a raised profile.

Needling is not an exact science, but reports of outcomes suggest that greater success is achieved with a lower IOP immediately after needling, performing a course of needlings (i.e. more than one if required), and use of sub-conjunctival anti-scarring mediations.^2, 3^

## If needling is appropriate, how am I going to do it?

As with any procedure, the results in your hands are what matter, not the minutiae of the technique compared to someone else. You want minimum complications and maximum success!

A good operative field is vital. Use of adequate anaesthesia (I use topical anaesthetic followed by sub-conjunctival lignocaine 2%), a speculum, vascular constriction (I use phenylephrine because it is handy in the clinic), and povidone iodine (or a similar preparation) make a world of difference.

Needling at the slit lamp has the advantage of immediate assessment of pressure effect and easy review at regular intervals after the procedure (more difficult when done in the middle of a busy operating list). Needling in the operating theatre allows you to proceed immediately to a more complex surgical intervention if necessary. Needles of size 25–30 g have been reported, as have MVR blades; some practitioners report being more adventurous in their choices. (I use a 30 g needle at the slit lamp and micro MVR in theatre.)

Most practitioners enter the conjunctiva at a distance from the area of scarring to be perforated and use a ‘slicing’ action to open a good-sized drainage channel (Figure 1). Plan your approach carefully and only work on the area of obstruction to flow.

The most common complication when needling is haemorrhage – either sub-conjunctivally or into the anterior chamber. If your view is obscured, then you should stop and try another time.

A hyphaema requires the patient to be reassured, as their vision will be affected: you should wait until you are sure the active bleeding has stopped. Let the patient rest for 30–60 minutes then check for ongoing haemorrhage and a pressure rise, since the blood can sometime obstruct drainage completely. Once stable, manage the patient as for a hyphaema post-trabeculectomy: discharge the patient and review within one week as appropriate.

The other post-needling complication is hypotony. I personally have only had to take one patient back to theatre for this to date. If the anterior chamber has significantly shallowed, let the patient rest and see if it reforms spontaneously. If it does, then manage the patient as for a low pressure following trabeculectomy. If it does not, then introducing viscoelastic or gas to the anterior chamber is your best option, with regular review as appropriate. Case reports exist of infection and mis-placed needles, but these are fortunately rare (hopefully because appropriate care has been taken by clinicians). Prophylactic topical antibiotics are used by most practitioners. Pre- and postoperative steroids remain a mainstay of therapy to prevent recurrent scarring.

Sub-conjunctival steroid and 5-fluorouracil (5FU) are the most common antiscarring preparations. Be extremely careful that the drugs do not enter the anterior chamber. If they do, wash out in theatre immediately. See page 75 for tips on administration of 5FU. Mitomycin C is being used more frequently, and interferon, sodium hyaluronate, and bevacizumab are amongst the many additional agents that have been reported, with varying success.

## If needling is not appropriate or has failed, what subsequent procedure is required?

This depends on all the above factors and what is possible in your unit. Cyclodiode ciliary body ablation, repeat trabeculectomy at a second site, formal revision of the existing trabeculectomy, and drainage tube implantation are the most common options.
